# Structure, membrane topology and influence of cholesterol of the membrane proximal region: transmembrane helical anchor sequence of gp41 from HIV

**DOI:** 10.1038/s41598-020-79327-6

**Published:** 2020-12-17

**Authors:** Christopher Aisenbrey, Omar Rifi, Burkhard Bechinger

**Affiliations:** 1 Institut de chimie de Strasbourg, UMR7177, University of Strasbourg/CNRS, 4, Rue Blaise Pascal, 67070 Strasbourg, France; 2 Institut Universitaire de France, Paris, France

**Keywords:** Biophysics, Structural biology

## Abstract

During the first steps of HIV infection the *Env* subunit gp41 is thought to establish contact between the membranes and to be the main driver of fusion. Here we investigated in liquid crystalline membranes the structure and cholesterol recognition of constructs made of a gp41 external region carrying a cholesterol recognition amino acid consensus (CRAC) motif and a hydrophobic membrane anchoring sequence. CD- und ATR-FTIR spectroscopies indicate that the constructs adopt a high degree of helical secondary structure in membrane environments. Furthermore, ^15^N and ^2^H solid-state NMR spectra of gp41 polypeptides reconstituted into uniaxially oriented bilayers agree with the CRAC domain being an extension of the transmembrane helix. Upon addition of cholesterol the CRAC NMR spectra remain largely unaffected when being associated with the native gp41 transmembrane sequence but its topology changes when anchored in the membrane by a hydrophobic model sequence. The ^2^H solid-state NMR spectra of deuterated cholesterol are indicative of a stronger influence of the model sequence on this lipid when compared to the native gp41 sequence. These observations are suggestive of a strong coupling between the transmembrane and the membrane proximal region of gp41 possibly enforced by oligomerization of the transmembrane helical region.

## Introduction

**Figure 1:**
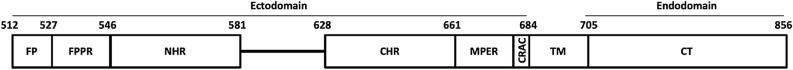
Schematic illustration of gp41: *FP* fusion peptide, *FPPR* fusion peptide proximal region, *NHR and CHR* N-terminal and C-terminal heptad repeat, respectively, *MPER* membrane proximal external region including the Cholesterol recognition amino acid consensus (CRAC) motif, *TM* transmembrane anchor, *CT* cytoplasmic terminus. The numbering follows the HXB2 subtype of HIV-1 (uniprot P04578).

Despite decades of intense research HIV remains a challenging infection that requires life-long treatment with expensive medicines and for which no effective vaccination has become available. During the HIV life cycle the viral and the target cell membranes have to fuse, a process mediated by the viral envelope gycoprotein *Env*^1,2^. The envelope protein is a heterodimeric complex of gp120 and gp41, these being the cleavage products of gp160. The gp41 protein forms a trimeric transmembrane unit and three gp120 proteins are exposed at the viral surface^3–5^. Interactions of gp120 with the cell surface receptor CD4^6^ and one of the chemokine receptors CCR5 or CXCR4^7–9^ initiates a series of conformational rearrangements during which gp120 is shed, thereby exposing gp41 which adopts an extended pre-fusion state^2,10^. The primary structure of gp41 is schematically illustrated in Fig. 1.

In the extended prefusion state the hydrophobic amino-terminal fusion peptide anchors in the host cell membrane while the gp41 transmembrane domain, which is 172 amino acid residues further downstream, remains in the viral envelope^10,11^. The presence of cholesterol in the viral and cellular membranes has been found important during membrane fusion, viral infection and the late phase of the viral cycle being a structural determinant and modulator of fusion and post-fusion events^12–14^.

Cholesterol is abundant in mammalian cells where is it is synthesized in the endoplasmatic reticulum, transported through the Golgi and accumulates at high concentrations in the plasma membrane. Cholesterol is also found in the blood serum in the form of lipoprotein complexes from where it exchanges with the cellular reservoirs (e.g. recent reviews^15,16^). Cholesterol has profound effects on the physico-chemical properties of lipid membranes where it orders lipids, increases the hydrophobic thickness of membranes, smoothens phase transitions, induces lateral lipid phase separations and membrane curvature strain^1^. In the presence of cholesterol, the formation of liquid-ordered (L_o_) and liquid-disordered phases (L_d_) has been observed, which are rich and poor in cholesterol, respectively^17–19^. More recent investigations revealed that the L_o_/L_d_ phase boundaries that occur when cholesterol-rich and cholesterol-poor membrane domains oppose each other is a key element for the functioning of gp41 fusion peptides^20,21^. Fusion works best when such boundaries occur in both the viral and the cellular membranes^22^.

Cholesterol has also been shown to interact with proteins either by changing the physico-chemical properties of the membrane or by direct interactions with amino acid side chains^1,23–25^. Thereby, in several high-resolution structures, cholesterol has been found to interact with the protein revealing a number of recurring contacts sites^25^. First, Ala, Leu and Val commonly interact with the isooctyl tail of cholesterol. Second, the sterol ring interacts with hydrophobic residues where the C18 and C19 methyl groups protrude out of the cholesterol ring and serve as knobs to fit holes or grooves on the protein surfaces predominantly involving branched residues such as Leu and Ile. Third, aromatic residues interact with the saturated parts of the sterol ring by van der Waals and CH–π interactions. Furthermore, π–π interactions of Phe and Tyr with the C5=C6 double bond have been observed. Fourth, the 3β-OH head group of cholesterol takes part in hydrogen bonding networks with water and polar amino acids in particular in loops and turns at the membrane interface^25^.

Indeed, combinations of the above mentioned amino acids have been found in motifs such as the ‘cholesterol recognition amino acid consensus’ (CRAC) domain (-L/V-X_1-5_-Y-X_1-5_-R/K-), the reversed CRAC motif (CARC), the cholesterol consensus motif (CCM), or the sterol sensing domain (SSD)^1,23–25^. However, it should be noted that statistically such combinations of amino acids occur quite frequently and, in most cases do not involve interactions with cholesterol^23,25^. Furthermore, the cholesterol consensus motif (CCM) has been identified in GPCRs and consists of amino acid arrangements involving several TM helical domains^26^ whereas the role of the sterol sensing domain (SSD) has been questioned^27^.

The membrane proximal external region (MPER) of gp41 is highly conserved among HIV and SIV strains^28^. The MPER, the fusion peptide and the heptad repeat sequences (Fig. 1) are all considered essential during the fusion of viral and cellular membranes by destabilizing the bilayer packing during fusion^1,29–32^. A CRAC motif with the sequence LWYIK^1,23–25,33^ is part of the membrane proximal region. Mutagenesis of CRAC resulted in a reduced infection rate and a slowing of the viral life cycle^34^. Notably, epitopes for the 2F5, 4E10, 10E8 and LN01 broadly neutralizing antibodies against HIV include locations of the MPER and knowing more about its structure bears considerable promise for the rational design of an AIDS vaccine^35–40^.

Whereas most studies are indicative of helical conformations of MPER in membrane environments considerable discrepancies exist about the details of how the domain interacts with lipid bilayers or how the associated transmembrane domain is organized^41–44^. For example, while the structure of the TMD has been evaluated from NMR data in DMPC/DHPC bicelles assuming a trimeric structure^45,46^, a more extensive analysis using NMR relaxation data, RDCs, PRE, EPR and ultracentrifugation data reveals that in such environments the TMD is a monomeric continuous helix extending into the MPER^43^. When a construct including a more extended MPER domain has been investigated the MPER amino-termini of three subunits form a hydrophobic core^44^. As a result the N- and C-terminal helices of MPER are connected by a 90° turn while the C-terminal helix adopts a kinked arrangement relative to the TMD. In yet another X-ray structure MPER has been found to form a continuous helix with the C-terminal heptad repeat sequence^47^. Notably, in the presence of 2F5 antibodies β-turn structures have also been observed for the MPER^48,49^.

The fusion events that follow the initial contacts of gp120 with the cellular receptors occur via transient structural rearrangements that remain largely speculative and result in a post-fusion structure where the fusion peptide and the transmembrane domain, although far removed in the primary sequence, are in close physical proximity^50^. In order to bring the membranes into contact the extended prefusion arrangement has to transform to the compact postfusion bundle of helical hairpins made of six helices in antiparallel arrangement^11,47,51^. The exposed pre-hairpin conformation is of particular interest because it is accessible to the humoral immune response during a relatively long lag phase of HIV infection^52,53^ and has become the target of antiviral fusion inhibitor drugs and vaccines^54–56^.

In order to understand the mechanism of viral entry into the host cells it is important to understand in molecular detail the structural changes of gp41 during receptor recognition, membrane fusion and cellular entry. Although high-resolution structural investigations promise to be excellent starting points to develop broadly neutralizing vaccines the conformational and topological details of the MPER domain remain mysterious. Structural data and propositions include an amphipathic helix localized in the membrane interface at an alignment parallel to the surface^29^, a helix-kink-helix structure where the helices are at about 45° relative to the membrane normal^44^ or a continuous extension of the transmembrane helix^43^. Notably, the most recent data from a plethora of MAS solid-state NMR distance and water accessibility measurements were obtained from bilayer samples and seem in accordance with a predictive theoretical model^29^ where a trimeric helix-kink-helix structure is obtained for a MPER-TMD construct^57^. When the data are used as constraints in evaluating structural templates from analogous viral fusion proteins the CRAC sequence constitutes part of the kinked non-helical region^57^. The diverging data suggest that the MPER region exhibits a high degree of conformational plasticity which may be of importance to optimize its interactions with membranes during the different stages of the viral life cycle.

Therefore, in this paper we have investigated polypeptide sequences encompassing the MPER connected to transmembrane helical membrane anchors and their interactions with cholesterol in liquid crystalline phospholipid bilayers by CD, FTIR and solid-state NMR spectroscopies. Whereas CD and FTIR spectroscopies reveal the secondary structure composition of polypeptides, solid-state NMR of polypeptides that are labelled with ^15^N and ^2^H at selected sites are sensitive indicators of interactions within membranes and provide direct information about the alignment of the labelled sites relative to the membrane normal^58^. In particular, we tested the structural and topological arrangement of the MPER, its structural coupling with the transmembrane helical anchor and if interactions of cholesterol with the CRAC domain of gp41 result in structural and/or topological changes.

## Materials and methods

Organic solvents were from Sigma-Aldrich (St Quentin Fallavier France) and of 99% purity. Cholesterol, POPC and POPS were from Avanti Polar Lipids (Birmingham, AL).

### Peptide synthesis

The peptides were prepared by solid-phase peptide synthesis using the standard cycles of a Millipore 9050 automatic peptide synthesizer (Millipore, Darmstadt, Germany) and Fmoc chemistry. Fmoc-protected amino acids from NovaBiochem (Merck Millipore, Darmstadt, Germany) were used at four-fold excess, the Tentagel-R-RAM resin was from Rapp Polymere (Tübingen, Germany). Cleavage of the peptide was performed in 28 mL trifluoroacetic acid (TFA), 1.5 mL water and 0.3 mL triethylsilane for 4 h. After solubilization and ether precipitation the TFA counter ions were exchanged in 4% acetic acid. After purification by semipreparative HPLC using an acetonitrile/water gradient on a C18 reverse-phase column (Luna 5u c18(2) 100 Å; 150 × 30 mm, Phenomenex, Le Pecq, France) the identity of the products was confirmed by MALDI TOF mass spectrometry.

### Preparation of samples for CD spectroscopy

The CRAC-TM_model polypeptide was dissolved in chloroform/methanol 2/1 v/v and mixed with a solution of lipid in the same solvent to obtain a peptide-to-lipid ratio of 1/100. CRAC-TM_gp41 was dissolved in trifluoro ethanol (TFE)/water 85/15 v/v and homogenously mixed with lipid in TFE/chloroform 1/1 v/v. The solvent was evaporated under a stream of nitrogen and in high vacuum overnight. The lipid film was then hydrated in 10 mM phosphate buffer, pH 7 to obtain a peptide concentration of 25 µM. Small unilamellar vesicles were prepared by tip sonication (Bandelin Sonoplus HD 200, Bandelin, Berlin, Germany) in an ice bath. Aggregates and titanium debris from the sonicator tip were removed by centrifugation at 10,000*g* for 5 min at room temperature.

### CD spectra

CD spectra were recorded on a JASCO-810 spectrometer (Jasco, Tokyo, Japan) at 20 °C between 190 and 250 nm with a band width of 1 nm. 4 scans of adaptive integration time between 1 and 8 s were averaged. A spectrum of buffer was subtracted. The spectra were analyzed for secondary structure composition of the peptides using CDPRO and the CONTIN/LL algorithm with reference set SMP56^59,60^.

### ATR FTIR measurements

For ATR FTIR measurements CRAC-TM_model samples were prepared by dissolving 1 mg of peptide in chloroform/methanol 2/1 v/v. Lipids (POPC, Cholesterol) in the same solvent were added to reach a peptide-to-lipid (P/L) molar ratio of 2%. A few µL of sample solution in 10 mM sodium acetate buffer prepared in D_2_O were deposited on the germanium crystal of a Nicolet 6700 ATR-FTIR spectrometer (Thermo Scientific, Waltham, MA). Typically, five ATR spectra, each of 128 scans, were recorded between 1000 and 1800 cm^−1^ with a resolution of 4 cm^−1^. The spectra were normalized to the amide I absorption band near 1655 cm^−1^ to facilitate their comparison and slightly smoothed by means of a binomial filter numerical procedure. In order to obtain the relative contribution of each secondary structure element, the deconvolution of the amide I signal is necessary (Origin 8.5 software).

### Preparation of supported lipid bilayers

The peptides were dissolved and mixed with lipids in the same solvents as used above (CD samples) to obtain a peptide-to-lipid ratio of 2 mol%. The solvents were evaporated under a stream of nitrogen until a viscous solution was obtained (~ 200 μL). The latter was applied to 22 ultrathin glass plates (8 × 22 mm or 6 × 11 mm, thickness 00, Marienfeld, Lauda-Königshofen, Germany) and dried first in air, then in high vacuum overnight. The glass plates were equilibrated in 93% relative humidity at ambient temperature, stacked on top of each other and sealed in plastic wrapping (illustrated protocol in^61^).

### Solid-state NMR spectroscopy

The samples were inserted into a flat-coil NMR probe^62^ with the normal parallel to the magnetic field direction of the NMR spectrometer.

Proton-decoupled ^15^N solid-state NMR spectra were recorded at 40.54 MHz on a Bruker Avance 400 solid-state NMR spectrometer (Bruker Biospin, Rheinstetten, Germany) at room temperature. A cross polarization (CP) pulse sequence^63^ was used with the following parameters: spectral width 37.5 kHz, acquisition time 3.5 ms, CP contact time 0.8 ms, recycle delay 3 s, B_1_ fields during CP 42 kHz. Typically, 40 k scans were accumulated. An exponential multiplication with line broadening of 100 Hz was applied before Fourier transformation. The spectra were calibrated relative to external ammonium chloride (40 ppm)^64^. The precision of the chemical shift determination depends on the line width and the signal-to-noise of the corresponding spectra, therefore, several samples have been recorded repeatedly. The data were analyzed by taking into consideration the angular dependence of the chemical shift σ_zz_ which is σ_zz_ = σ_11_ sin^2^Θ cos^2^Φ + σ_22_ sin^2^Θ sin^2^Φ + σ_33_ cos^2^Θ. In this formula σ_11_, σ_22_ and σ_33_ are the main tensor elements^64^, and the Euler angles Θ and Φ position the chemical shift tensor relative to the magnetic field direction of the NMR spectrometer^65^.

^2^H solid-state NMR spectra were recorded at 61.4 MHz on a Bruker Avance 400 solid-state NMR spectrometer (Bruker Biospin, Rheinstetten, Germany) at room temperature. A quadrupolar echo pulse sequence was used^66^ with a spectral window, an acquisition time, echo delay, and a recycle delay of 125 kHz, 6.8 ms and 1.5 s, respectively. The echo delay was twice 40–50 μs. Typically, 40 k scans were accumulated. An exponential multiplication with line broadening of 300 Hz was applied before Fourier transformation. D_2_O was used as an external reference.

## Results

### Peptide design

Domains considered important during the viral life cycle were prepared by solid-phase peptide synthesis and HPLC purification in order to investigate their membrane interactions. During the synthesis ^15^N and ^2^H isotopic labels were included into the sequences for solid-state NMR structural investigations. The sequences thus prepared and investigated are listed in Table 1. The CRAC sequence of gp41 was anchored at the membrane interface by its covalent linkage to a transmembrane anchor. The membrane anchor is either the wild type sequence of gp41 which has been shown to adopt helical conformations in previous investigations^43–45^ (noted: *TM_gp41*), or a helical hydrophobic model peptide made of alanines and leucines similar to those investigated in previous studies^67–69^ (*TM_model*). As for the CRAC sequence, the native LWYIK was incorporated and its interactions compared to those of a scrambled sequence (*scr*) which does not follow the published amino acid consensus motif^33^. Thereby it is possible to distinguish if chemical shift changes upon addition of cholesterol are due to peptide-lipid interactions or rather reflect alterations in the physico-chemical properties of the membrane. Furthermore, an I/L variant was investigated which also fits the CRAC motif (*model_2*). Finally, the position of the ^15^N label was varied to test if the topological findings at Leu 8 are reproduced at Leu 11 thereby reflecting the CRAC domain as a whole (^*15*^*N-L8* or ^*15*^*N-I11*).

**Table 1:**
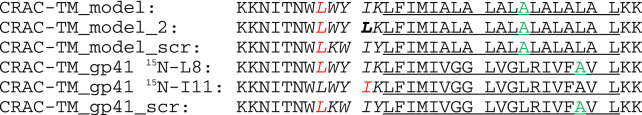
Peptides derived from gp41 and investigated in this paper (*Env* HV1H2 UniProtKB–P04578; residues 674–702 or parts thereof).

The CRAC motif is shown in italics, the transmembrane domain is underlined. ^15^N labelled sites are shown in red, ^2^H_3_-alanines in green. The substituted leucine of the CRAC variant is shown in bold.

#### Secondary structure in lipid bilayers by CD- and FTIR spectroscopies

In a next step the secondary structure of the polypeptides in membrane environments was investigated by CD spectroscopy. The hydrophobic anchoring sequence and the lipid reconstitution protocol starting from a homogenous solution of peptides and lipids in organic solvent assure that virtually all of the polypeptide is associated with the liposomes. The polypeptides were designed in such a manner to adopt a high degree of helical conformation in membranes. Thereby, the spectroscopic investigations also provide a first test if the peptide design and the membrane reconstitution protocol worked as expected.

**Figure 2:**
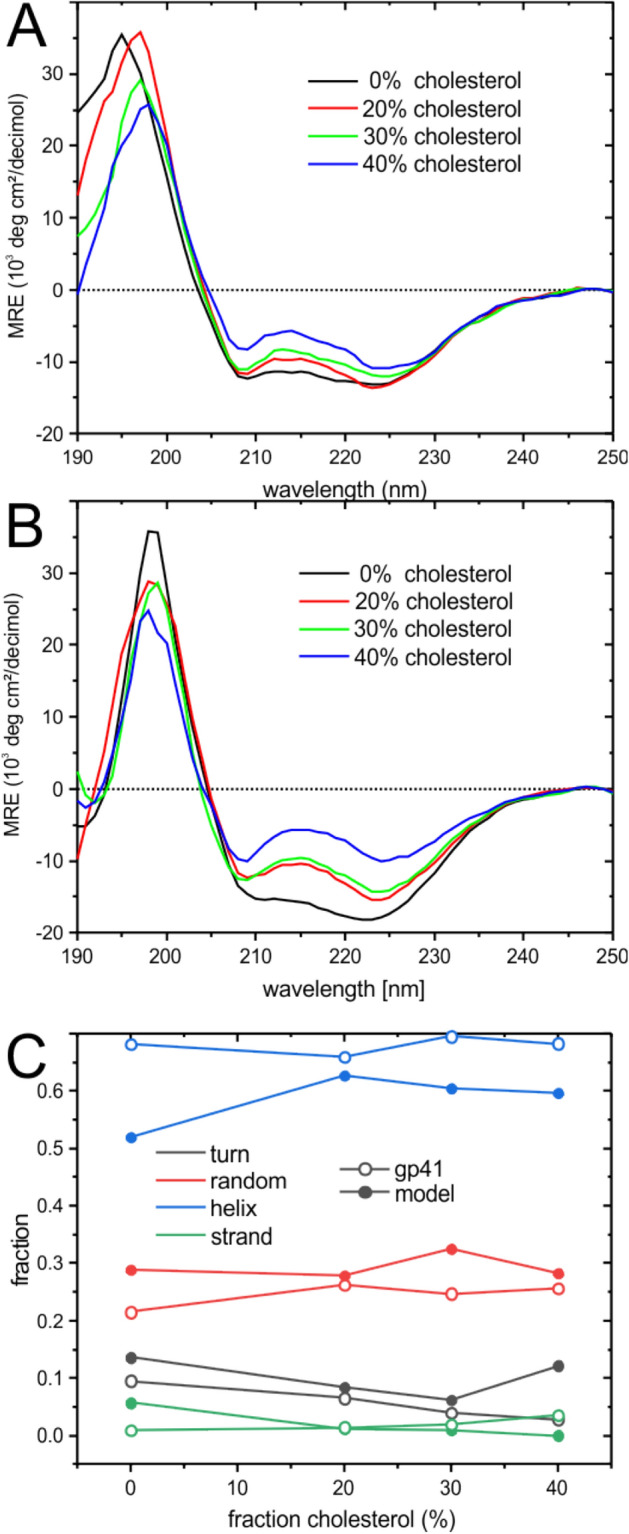
CD spectra of (**A**) CRAC-TM_model and (**B**) CRAC-TM_gp41 after reconstitution into POPC/POPS 3/1 mol/mole SUVs with increasing concentrations of cholesterol at a peptide-to-lipid ratio of 1 mol%. The peptide concentration was 25 µM in 10 mM phosphate buffer, pH 7, at 20 °C. (**C**) Fit results of the CD spectra of CRAC-TM_model (closed symbols) and of CRAC-TM_gp41 (open symbols) using the CONTIN/LL algorithm and the base set SMP56 of CDPRO. Comparison of the experimental spectra and the fits are shown in Figure S1.

The CD spectra of CRAC-TM_model or of CRAC-TM_gp41 exhibit the typical features of helical polypeptides when investigated in POPC/POPS 3/1 mol/mole membranes, i.e. a positive maximum at 195 nm and two negative intensities at 208 nm and 222 nm (Fig. 2). Indeed, line fitting analysis using the Dichroweb software indicates helical contributions > 50%^59,60^. At higher cholesterol concentrations the spectra flatten and a more quantitative analysis has to be considered with caution because of light scattering artifacts in the presence of vesicles of a size approaching the wave length of the incoming light^70^. Therefore, the fit results shown in Fig. 2C should be considered semiquantitative.

**Figure 3:**
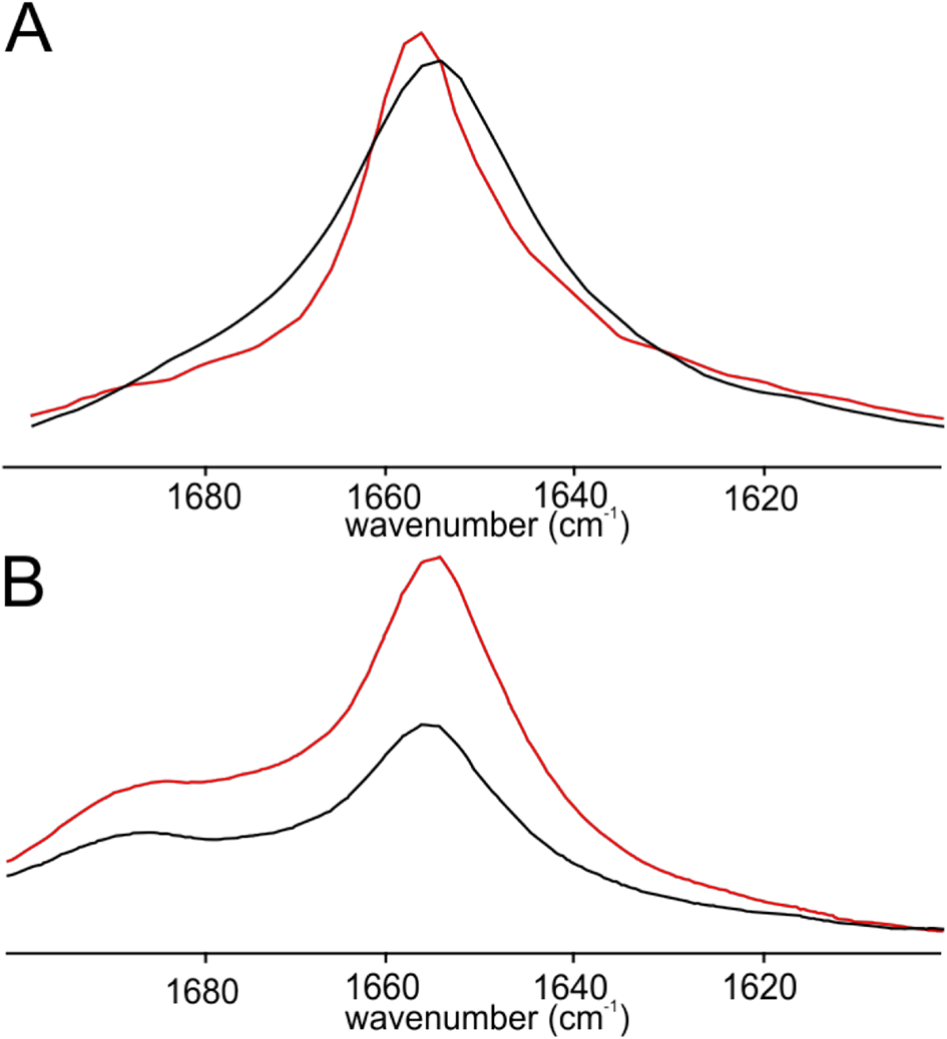
ATR- FTIR spectra of (**A**) CRAC-TM_model and (**B**) CRAC-TM_gp41 reconstituted into POPC (black) or POPC/cholesterol 70/30 mol/mole membranes (red).

Because peptide oligomerization/aggregation and vesicle agglutination can have a pronounced effect on the CD spectra, when at the same time they depend on all of lipid composition, preparation protocol, exact P/L ratio and lipid concentration, the secondary structure preferences were further investigated by ATR-FTIR spectroscopy where a lipid suspension deposited on a solid support is investigated. In contrast to CD spectroscopy light diffraction artefacts due to the agglutination and/or fusion of vesicles are avoided. The ATR-FTIR spectra of CRAC-TM_model reconstituted in POPC or POPC/cholesterol 70/30 mol/mole lipid bilayers exhibit a single resonance with a peak position characteristic of helical conformations (Fig. 3A)^71^. In contrast CRAC-TM_gp41 shows additional intensities > 1665 cm^−1^ characteristic for β-turn conformations (Fig. 3B).

### Solid-state NMR investigations of the CRAC domain in oriented lipid bilayers

Previous structural studies have come to different conclusions with regard to the membrane interactions of the MPER which includes the CRAC domain^57^. The data are suggestive of an orientation along the surface^29^, a helix-kink-helix structure where the helices are at about 45° relative to the membrane normal^44^ or a continuous extension of the transmembrane helix^43^. Whereas such previous structures were calculated from a large number of short range NMR parameters static oriented solid-state NMR spectroscopy is a complementary approach based on bond orientations relative to the membrane derived from the anisotropy of chemical shift, dipolar and quadrupolar interactions^58,65^.

Therefore, in order in investigate in more detail the interactions of the CRAC-TMD polypeptides with lipid bilayers, and in particular to get direct insight into the alignment of the CRAC sequence relative to the membrane surface, the peptides were prepared carrying isotope labels at specific sites, reconstituted into supported liquid crystalline phospholipid bilayers and solid-state NMR spectra were recorded. When inserted into the solid-state NMR probe with the sample normal parallel to the magnetic field direction the resulting ^15^N chemical shift of the peptide amides correlates in a direct manner with the alignment of the amide ^15^N-^1^H vector and thereby the helical tilt angle^65^. Whereas ^15^N–^1^H bonds that are oriented parallel to the membrane normal (i.e. transmembrane helices) exhibit ^15^N chemical shifts around 200 ppm those that are oriented along the surface resonate at < 100 ppm. Finally, unstructured domains either aggregate and show broad powder pattern line shapes (50–230 ppm) or isotropic peak positions (ca 120 ppm)^72^.

**Figure 4:**
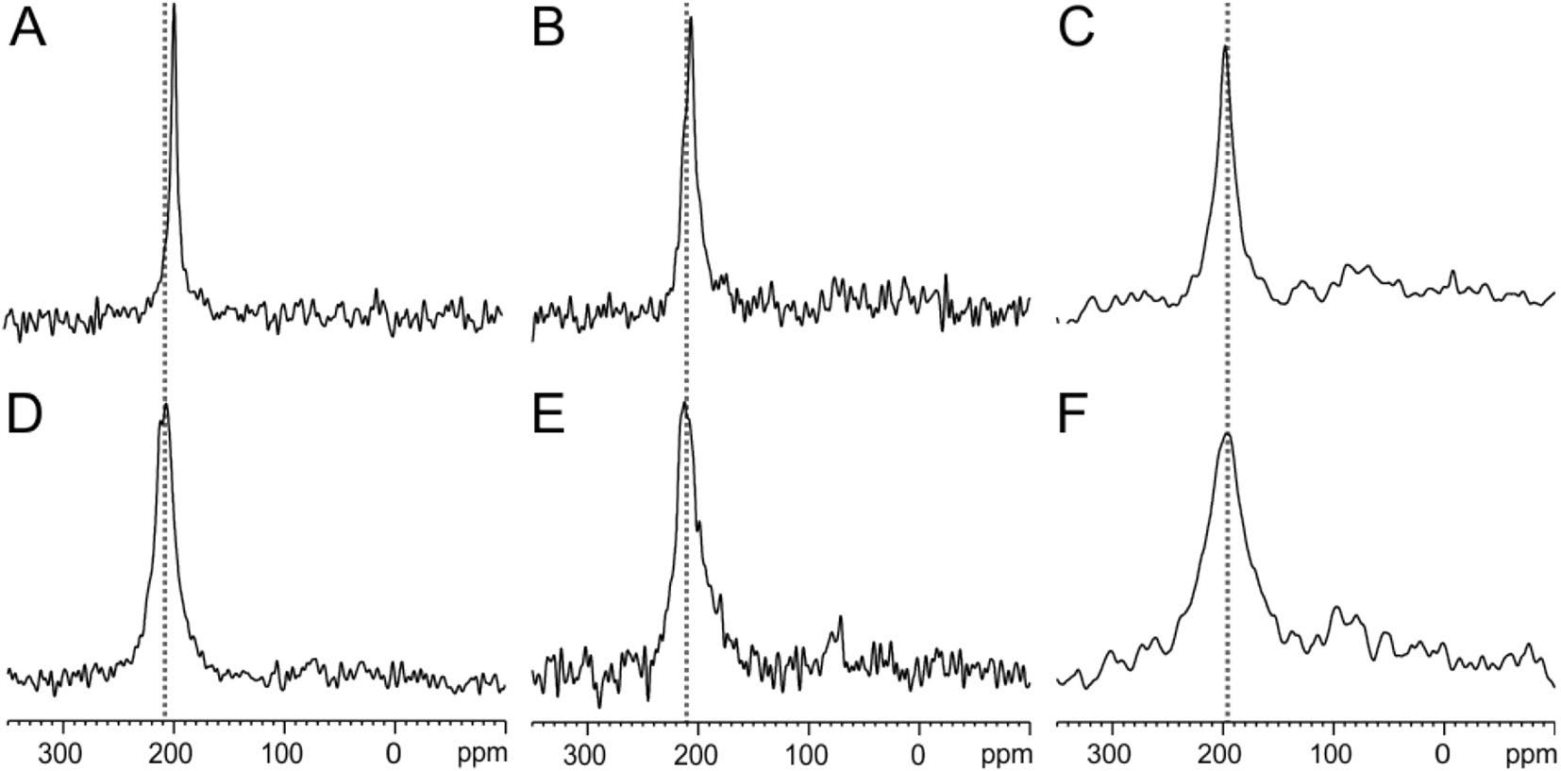
Proton-decoupled ^15^N solid-state NMR spectra of [^15^N-Leu8]-CRAC-TM_model (**A**,**D**) [^15^N-Leu8]-CRAC-TM_model-scr with a scrambled amino acid arrangement of the CRAC region (**B**,**E**) and of [^15^N-Leu8]-CRAC-TM_gp41 (**C**,**F**). The polypeptides were reconstituted into uniaxially aligned POPC lipid bilayers without (**A**–**C**) and with 30 mol% cholesterol (**D**–**F**) at a peptide-to-lipid ratio of 2 mol%. The membrane normal was parallel to the magnetic field of the NMR spectrometer and the experiments were performed at room temperature.

In a first set of experiments the CRAC-TM_model sequence labelled at the Leu8 position, i.e. within the CRAC motif, was investigated (Fig. 4, Table 2). The spectra show well-oriented ^15^N solid-state NMR spectra in the 200 ppm region which are typically observed for transmembrane helical sequences. In POPC the chemical shift is 197 ppm which changes to 208 ppm upon addition of 30 mol% cholesterol (Fig. 4A,D). Closely related spectra are obtained for CRAC-TM_model_2 which only differs in a single I-L substitution (Tables 1 and 2, Figure S2). When the CRAC sequence is scrambled the chemical shift is about 210 ppm in the absence or presence of cholesterol (Fig. 4B,E, Table 2). For all three peptides, in the presence of cholesterol the peaks become broader by about two-fold suggesting that in the cholesterol containing membrane motional averaging is less dynamic^1^.

**Table 2:** ^15^N chemical shift measurements of [^15^N-Leu8]-CRAC-TM_model labelled with ^15^N within the CRAC sequence, reconstituted into POPC lipid bilayers that were uniaxially oriented with the normal parallel to the magnetic field direction at peptide-to-lipid ratios of 2 mol% in the absence or presence of 30 mol% cholesterol.

Sequence	^15^N chemical shift (ppm)		LWHH (ppm)	
	30% cholesterol		30% cholesterol	
	Without	With	Without	With
CRAC-TM_model	196.5 ± 2.2	208 ± 6.5	12.5	25
CRAC-TM_model_2	197.5 ± 2.5	208 ± 6	15	30
CRAC-TM_model-scr	209 ± 4.5	211 ± 7	20	30

The errors in determining the chemical shift were derived from the spectral resolution, the line width and the signal-to-noise ratio.

*LWHH* line width at half height.

In a second series of experiments the CRAC-TM_gp41 sequence was labelled at the Leu8 or the Ile11 position, reconstituted into oriented membranes and investigated in the presence of 0, 2, 10 or 30 mol% cholesterol (Fig. 4C,F, Table 3). Irrespective of the cholesterol content, ^15^N chemical shifts around 198 ppm were observed for Leu8, and at about 215 ppm for Ile11. Although the chemical shift position did not change significantly with the addition of cholesterol the line width at half-height increased from 15 to 40 ppm for Leu8 (Fig. 4C,F, Table 3) and from 7 to 14 ppm for Ile11 (Table 3).

**Table 3:** ^15^N chemical shift measurements of CRAC-TM_gp41 labelled with ^15^N within the CRAC sequence, reconstituted into POPC lipid bilayers that were uniaxially oriented with the normal parallel to the magnetic field direction at peptide-to-lipid ratios of 2 mol% in the presence or absence of cholesterol.

Label	^15^N-Leu8		^15^N-Ile11	
% Cholesterol	^15^N chemical shift (ppm)	LWHH (ppm)	^15^N chemical shift (ppm)	LWHH (ppm)
0	199.5 ± 1.5	15	213.5 ± 2.2	7
2	197.5 ± 5	20	216 ± 2.2	9.5
10	195.5 ± 2.5	17.5	215.4 ± 2.3	8
30	197.5 ± 9	40	213 ± 4.3	14

The errors in determining the chemical shift were derived from the spectral resolution, the line width and the signal-to-noise ratio.

*LWHH* line width at half height.

### Solid-state NMR investigations of the TM domain in oriented lipid bilayers

Whereas ^15^N solid-state NMR investigations provide data on the membrane alignment and interactions of the CRAC domain (Fig. 4, Tables 2 and 3) the membrane interactions of the TMD are analyzed by recording ^2^H solid-state NMR spectra from the same samples. For these experiments ^2^H_3_-alanine was incorporated at position 24 of the CRAC-TM_model or position 29 of the CRAC-TM_gp41 sequences. The deuterium labels thus represent the situation within the transmembrane anchor. Previous NMR investigations indicate a stable helical conformation of the gp41 membrane anchor when studied in bicellar environments^43–45^. Furthermore, hydrophobic model sequences that are closely related to the one investigated here and carrying isotopic labels at comparable positions exhibit solid-state NMR spectra typical of stable helical structures in liquid crystalline bilayers^67–69^.

**Figure 5:**
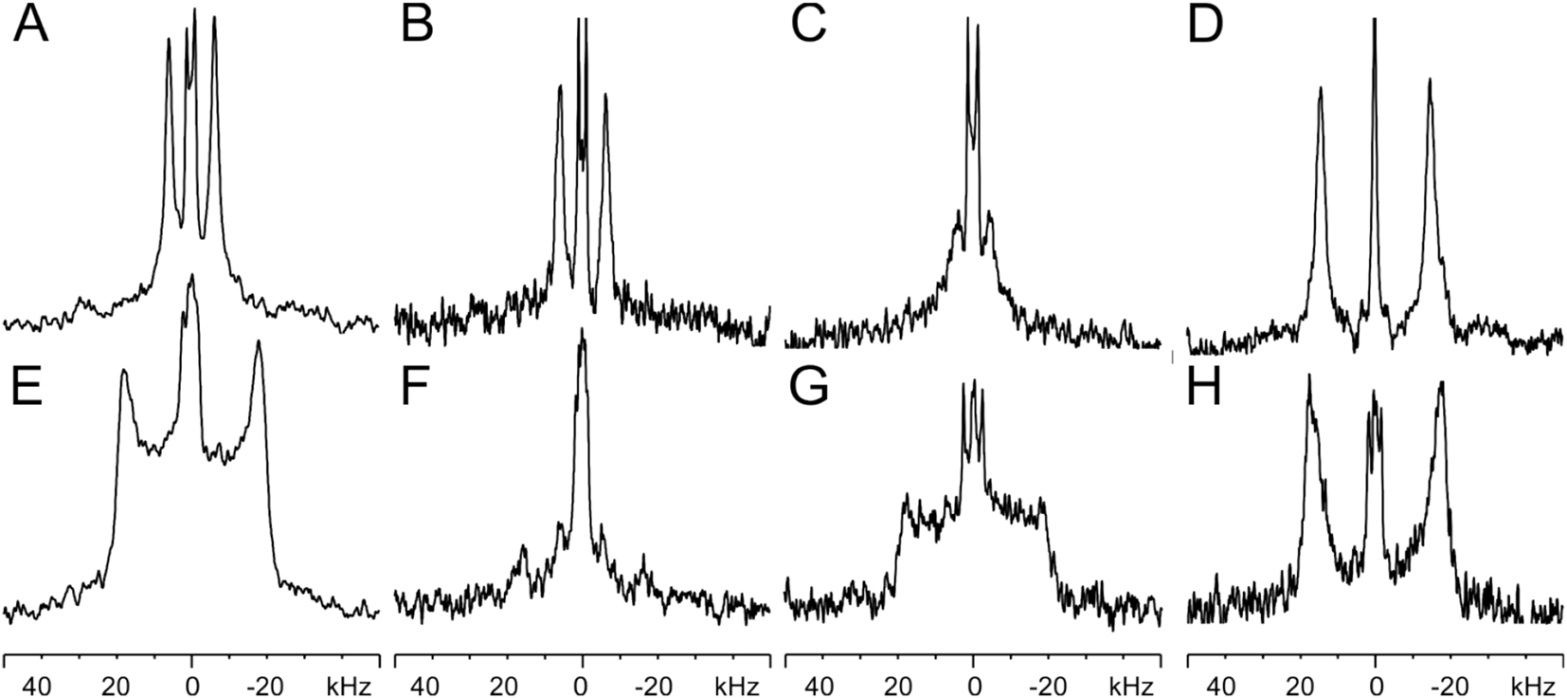
^2^H solid-state NMR spectra of 2 mol% [3,3,3-^2^H_3_-Ala24]-CRAC-TM_model (**A**,**E**), [3,3,3-^2^H_3_-Ala24]-CRAC-TM_model2 with a single I to L substitution within the CRAC domain (**B**,**F**), [3,3,3-^2^H_3_-Ala24]-CRAC-TM_model with a scrambled CRAC amino acid arrangement of the CRAC domain (**C**,**G**) and of [3,3,3-^2^H_3_-Ala29]-CRAC-TM_gp41 reconstituted into uniaxially aligned POPC lipid bilayers (**D**,**H**) without (**A**–**D**) and with cholesterol (**E**–**H**). The membrane normal was parallel to the magnetic field of the NMR spectrometer and the experiments were performed at room temperature. The central contributions exhibiting small quadrupolar splittings and isotropic resonances are from residual HDO^74^.

Notably, in POPC membranes well-oriented ^2^H solid-state NMR spectra are observed for all four sequences i.e. the CRAC-TM_model carrying the native CRAC, the scrambled CRAC (CRAC-TM_model_scr) or the I11L substitution CRAC as well as the CRAC-TM_gp41 sequence (Fig. 5A–D, Table 4). It should be noted that relatively narrow angular distributions of only 5° or 10° can result in considerable line broadening of the ^2^H solid-state NMR spectra recorded from deuterated alanines^68,73^. In contrast the ^15^N spectra of backbone labelled transmembrane helices are much less sensitive to angular variations^65^. Because the ^2^H quadrupolar splitting is so sensitive to even small orientational changes^58,68^ this indicates that in POPC the TMDs exhibit unique membrane alignments with very little mosaicity.

**Table 4:** ^2^H quadrupolar splittings of [^2^H_3_-Ala]-labelled CRAC-TM sequences where the isotopic label is within the transmembrane domain, reconstituted into POPC lipid bilayers that were uniaxially oriented with the normal parallel to the magnetic field direction at peptide-to-lipid ratios of 2 mol% in the presence or absence of 30 mol% cholesterol.

Sequence	^2^H quadrupolar splitting (kHz)	
	Cholesterol	
	0%	30%
[^2^H_3_-Ala24]-CRAC-TM_model	12	0–30^a^
[^2^H_3_-Ala24]-CRAC-TM_model 2	12	0–30^a^
[^2^H_3_-Ala24]-CRAC-TM_model-scr	9	0–35^a^
[^2^H_3_-Ala29]-CRAC-TM_gp41	30	35

^a^Indicates a broad signal covering a range of quadrupolar splittings.

When 30% cholesterol is added to the POPC bilayers the spectra of the [3,3,3-^2^H_3_-Ala24]-model sequence broaden considerably (Fig. 5E–G) whereas the gp41 transmembrane domain remains largely unaffected (Fig. 5H, Table 4). The broad distribution of quadrupolar splittings covering ≤ 35 kHz is indicative of an increased range of conformations and/or topologies that result in C_α_–C_β_ orientations in slow (Fig. 5E,G) or intermediate exchange (Fig. 5F) on a time scale of 10^–5^ s.

In contrast, a single alignment persists for the gp41 TMD with little topological heterogeneity (Fig. 5H). In the presence of cholesterol, the quadrupolar splitting of [3,3,3-^2^H_3_-Ala29]-CRAC-TM_gp41 changes by 5 kHz (Table 4) which is indicative of a change in C_α_–C_β_ orientation by a few degrees and/or a decrease in motional averaging.

### Solid-state NMR spectra of deuterated cholesterol

**Figure 6:**
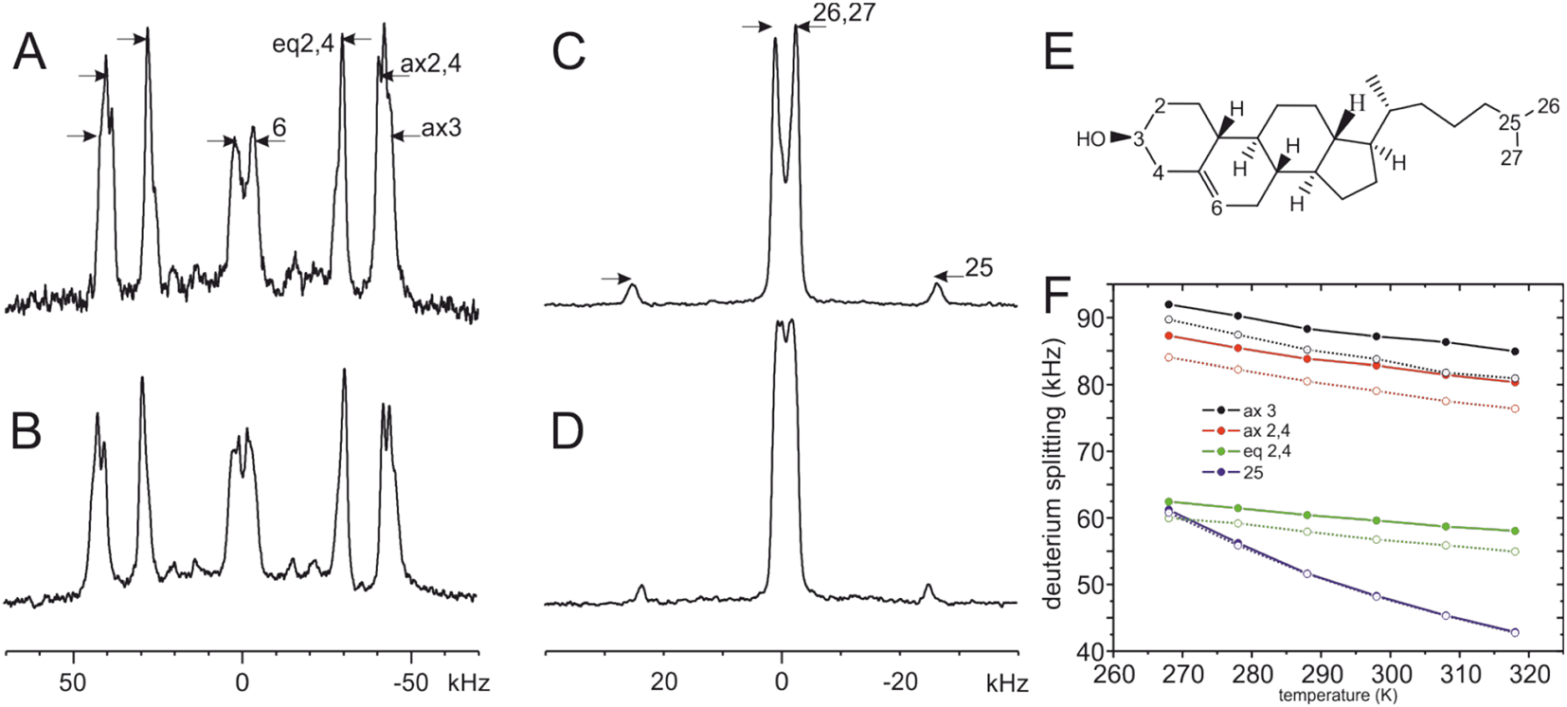
Deuterium solid-state NMR spectra of [2,2,3,4,4,6]-^2^H_6_-cholesterol (**A**,**B**) or [25, 26_3_, 27_3_]-^2^H_7_-cholesterol (**C**,**D**) in the absence (**A**,**C**) or presence (**B**,**D**) of 2 mol% CRAC-TM_gp41 in POPC/cholesterol 90/10 mol/mole membranes. (**E**) Structure of cholesterol where the deuterated sites are numbered. (**F**) The deuterium quadrupolar splittings extracted from the corresponding ^2^H solid-state NMR spectra as a function of temperature in the absence (solid lines, closed symbols) or presence of 1 mol% CRAC-TM_model (hatched line, open symbols). Positions 2 and 4 carry two deuterons each with different alignments (axial and equatorial relative to the ring system) and therefore different quadrupolar splittings.

Whereas in the experiments just presented the effects of cholesterol on the structure and alignment of the CRAC-TM sequences were tested (Figs. 4 and 5, Tables 2, 3 and 4) a complementary view on these interactions is obtained from NMR spectra of the surrounding lipids. Therefore, in the next series of experiments, the ^2^H solid-state NMR spectra of deuterated cholesterol were recorded in order to monitor its interactions with the CRAC-TMD sequences in liquid crystalline bilayers. Cholesterol was deuterated at either six sites at the 2, 3, 4 and 6 positions, which are expected to locate in the interfacial region in mixed phospholipid/cholesterol membranes, or at seven sites of carbons 25, 26 and 27 which reside in the hydrophobic membrane interior (Fig. 6E). The ^2^H solid-state NMR spectrum of a pure lipid membrane made of POPC/cholesterol-d_6_ 90/10 mol/mole is shown in Fig. 6A and compared to the spectrum obtained in the presence of 2 mol% CRAC-TM_ gp41 (Fig. 6B). Furthermore, Fig. 6C,D show the ^2^H solid-state NMR spectra of POPC/cholesterol-d_7_ 90/10 mol/mole in the absence or presence of this polypeptide, respectively.

In the presence of 2% CRAC-TM_gp41 a small increase in the quadrupolar splittings is observed for membranes enriched with 10% cholesterol-d_6_ (positions 2,3,4,5,6) when investigated at ambient temperatures (Fig. 6A,B). The same changes are observed in the presence of CRAC-TM_gp41_scr carrying a scrambled CRAC motif (not shown) suggesting that these alterations are unrelated to specific interactions with the cholesterol recognition motif.

Furthermore, a small decrease of the splittings assigned to the CD label at position 25 as well as of the CD_3_ groups at positions 26 and 27 occurs in the presence of 2% CRAC-TM_gp41 (Fig. 6C,D). Interestingly, the splittings of the methyl groups not only decrease but also split up into two distinguishable contributions upon addition of CRAC-TM_gp41 (Fig. 6C,D) suggesting a decrease in molecular order parameter and/or conformational changes of the cholesterol alkyl chain.

When the interactions of CRAC-TM_gp41with cholesterol were investigated neither the ^15^N chemical shifts of the peptide (Figs. 4C,F, Table3) nor the ^2^H spectra of deuterated cholesterol (Fig. 6A–D) showed any changes that would indicate a strong and specific interaction between the CRAC motif and the lipid. Because a small ^15^N chemical shift change due to the presence of cholesterol was observed for the CRAC motif when associated with the transmembrane model sequence (CRAC-TM_model; Fig. 4A,D), we performed additional measurements by reconstituting this peptide into membranes carrying deuterated cholesterol (Figs. 6F and S3).

The temperature dependent decrease of the quadrupolar splittings of the various sites in the presence of CRAC-TM_model is shown in Fig. 6F where the assignment has been taken from previous publications^75,76^. The comparison shows that the quadrupolar splittings of the ^2^H-labels at the 2, 3 and 4 positions decreases by about 4% due to the presence of CRAC-TM_model, while the deuteron at the 25 position does not show any difference due to the incorporation of this polypeptide (Figs. 6F and S3). Because the C^2^H_3_-signals of the 26 and 27 positions exhibit a small splitting they potentially overlap with residual HDO resonances and have not been analyzed further.

## Discussion

Because in previous structural investigations ambiguous data have been obtained about the membrane alignment and interactions of the CRAC sequence (reviewed in reference^57^), here we have used oriented solid-state NMR to obtain angular information on several labelled sites in the presence and absence of cholesterol. In contrast to the distance information obtained by solution and MAS solid-state NMR from which the membrane topology of protein domains is deduced in an indirect manner^29,43,44,57^ solid-state NMR spectroscopy on supported lipid bilayers works in a complementary way by directly measuring angular information of peptide chemical bonds relative to the membrane normal^58,65^.

The CRAC motif of gp41 was positioned at the membrane interface via transmembrane helical anchor sequences, and its structural response to cholesterol was tested using CD- and FTIR- as well as oriented solid-state NMR spectroscopies. The mutual influence of the transmembrane helical domain and the MPER was investigated by replacing the native gp41 sequence by a simple hydrophobic model anchor. Furthermore, control experiments consisted in a scrambled CRAC sequence, a variant of the CRAC motif and sequences that carried the isotopic labels at different positions (Table 1).

### Secondary structure and membrane topology of CRAC-TM

In a first series of experiments the constructs were studied for secondary structure preferences. As expected from the experimental design CD- and FTIR spectra were dominated by helical contributions in the presence or absence of cholesterol (Figs. 2 and 3). Interestingly, the FTIR technique reveals some β-turn contribution for the polypeptides anchored with the native gp41 TMD (Fig. 3B). Helical preferences in the presence of DPC micelles were also found when peptides presenting the pre-transmembrane region of gp41 were investigated by solution NMR-, CD- and infra-red spectroscopies^29^ while a ^13^C solid-state NMR chemical shift analysis revealed a tendency for the carboxy-terminal part of the gp41 TMD to adopt some β-sheet character in complex cholesterol-containing membranes mimicking the HIV envelope^57^.

**Figure 7:**
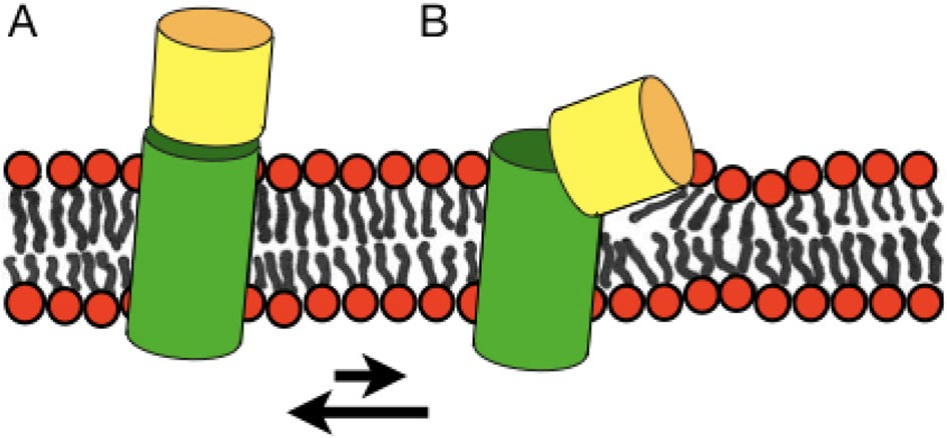
Sketches the conformational flexibility of gp41 where the TMD shown in green and the MPER/CRAC regions shown in yellow exhibit predominantly helical structures. The protein appears to exhibit considerable conformational flexibility. Whereas the data shown in this paper indicate that both helical domains are aligned in the same direction, possibly forming a continuous structure (**A**), helix-kink-helix conformations have also been observed (**B**). Possibly, the two conformers shown represent different states of the membrane fusion process (see text for details).

When the CRAC motif LWYIK was labelled with ^15^N at the Leu or the Ile position and the resulting CRAC-TMD constructs incorporated into uniaxially oriented POPC lipid bilayers the ^15^N spectra exhibit well oriented line shapes with chemical shifts around 200 ppm typically observed for helical domains that are aligned with the helix axis parallel to the membrane normal (Figs. 4 and 7A)^65,77,78^. Whereas such spectra have often been observed for hydrophobic transmembrane regions the CRAC sequence is part of the MPER. Therefore, the spectra are in line with a continuous helical arrangement as has been observed for a related gp41 sequence in bicellar environments by solution state NMR^43^. A continuous CRAC-TMD helical conformation has also be observed in the MAS solid-state NMR structure of TSPO^79^. Homology modelling with the crystal structures of bacterial counterparts indicated an ≈ 7° change in tilt angle of two helices upon interaction with cholesterol^79^. While the CRAC domain itself tends to stick into the water phase (Fig. 7A) comparison with other structures are suggestive that this configuration is not exclusive and can be affected by interaction contributions from upstream residues that pull the membrane external residues closer to the membrane^29,57^ (Fig. 7B) or stabilize a helix-kink-helix structure involving the CRAC motif^44^.

### Differential effects of cholesterol on CRAC-TM structure and topology

When the effect of adding 30% cholesterol to CRAC-TM_model reconstituted into supported POPC lipid bilayers is investigated the ^15^N chemical shift of the labelled Leu increases from 197 to 208 ppm (Table 2), which corresponds to a topological change in tilt/pitch angles by a few degrees^65^. Notably, the change was absent in presence of the scrambled CRAC sequence (Table 2). Thereby the spectral alterations point to an interaction between the CRAC motif and cholesterol, albeit when CRAC is attached to TM_gp41 such changes are absent (Table 3). Interestingly, when sub-nanometer proximities between cholesterol and the somewhat more extended MPER-TM_gp41 were tested in a recent MAS solid-state NMR investigation it turned out that cholesterol is close to two hot spots made up residues 673–677 and 684–688 but not in proximity of residues 679–683 which make up the CRAC motif^80^. In combination with the results of this paper this data suggests a weak interaction of cholesterol with the CRAC motif which can be overruled in a competitive manner by interactions with other domains. In this context it is of interest that residues 683–689 of TM_gp41 cover a CARC sequence^80^.

To gain further insight into the polypeptide topology the transmembrane domains were labelled with ^2^H_3_ at one of the alanine methyl groups and investigated by ^2^H solid-state NMR. This approach has previously been demonstrated to be highly sensitive to even small changes of the relative alignment of the alanine C_α_–C_β_ bond with respect to the membrane normal^58^. The addition of cholesterol has only a small effect on the wt TM_gp41 whereas a range of C_α_–C_β_ alignments is observed for the TM_model sequence (Fig. 5, Table 4). The broadening of the ^2^H NMR signal in case of TM_model can reflect conformational heterogeneity and/or many different topologies in slow exchange. It is possible that TM_model scans different alignments also in pure POPC bilayers but that in this lipid environment exchange is fast thus only an average orientation becomes apparent on the time scale of the ^2^H solid-state NMR spectra (10^–5^ s). In contrast, such motions are slowed sufficiently to make them distinguishable in the presence of cholesterol.

When the inverse experiment is performed where unlabeled peptide is added to deuterated cholesterol only in the presence of CRAC-TM_ model a significant reduction in the deuterium quadrupolar splittings of the interfacial cholesterol deuterons was observed whereas CRAC-TM_gp41 or the scrambled CRAC motif did not have an effect (Fig. 6). Thus, interactions between CRAC-TM_gp41 and cholesterol do not result in significant structural changes of either interaction partner (Tables 3 and 4, Fig. 6).

Interestingly, SDS gel electrophoresis is indicative of higher molecular weight oligomers formed by CRAC-TM_gp41 when compared to CRAC-TM_model (Figure S4). Therefore, the observed differences could be related to the association of the native TMD into oligomers (probably trimers)^57^ which is less likely for the model sequence. Thereby, within the oligomeric assembly of the wild-type sequence structural and topological changes of the CRAC or TM domains are energetically too costly to be triggered by the relatively weak interactions with cholesterol^57^. Indeed, the gp41 TMD carries a GxxxG motif (amino acids 19–23 of CRAC-TM_gp41) as well as an arginine at position 25, both being known to drive helix-helix association within the hydrophobic environment of the membrane interior^81–84^.

### Possible cholesterol recognition sites within gp41

Although the sequence and interfacial localization of the CRAC fit to the requirements for interaction with cholesterol it has been shown that there is a high statistical probability to find an amino acid arrangement fulfilling the criteria of a CRAC motif^23,25^. Thus, the motif has been found at high abundance in the proteins of a cholesterol-free bacterium^85^. Furthermore, of 19 high-resolution crystallographic structures where cholesterol has been detected a single carrier protein but none of the membrane proteins interacted with cholesterol via the CRAC or CARC motifs^25^. It is interesting to note that recent solid-state NMR studies of MPER- gp41_TMD revealed that cholesterol is localized too far from the membrane external CRAC motif to efficiently interact with this region^80^. Furthermore, the Influenza virus hemagglutinin M2 TMD tetramer revealed interactions of cholesterol with a hydrophobic cleft but not the CRAC sequence of this protein^76,86^.

When membrane protein structures which include cholesterol^25^ are investigated the cholesterol molecules are aligned parallel to the helical long axes with the hydroxyl being situated within the interface (e.g. pdb 4HYT at the level of the lipid phosphate). In this context it is interesting to note that the CRAC-TM_model sequence has an influence on the deuterium labels at positions 2, 3 and 4 of cholesterol but did not affect the deuterium NMR spectra of the 25-position of cholesterol confirming that the interactions between protein and lipid are more pronounced within the interface (Fig. 6). Because here we found that the membrane external CRAC motif (residues 679–683) is an extension of the TM helix it is probably positioned too far out for efficient direct interactions with cholesterol (Fig. 7A) even though the bilayer hydrophobic thickness increases by 4 Å in the presence of 30% cholesterol^87^. Thus, the weak interactions between cholesterol and the gp41 constructs investigated here probably involve other amino acid side chains than the CRAC motif.

For example, the model TMD starts with KLFIMIALA which positions Leu and Ile residues close to the interface (Table 1). These residues have been shown to be involved in knobs-into-hole interactions with the C19 methyl of cholesterol^25^. Furthermore, Phe residues have been shown to interact with the C5=C6 double bond by π–π interactions, and finally Lys and Met could establish a hydrogen bond with the cholesterol OH^25^. As for the gp41 TMD sequence KLFIMIVGG similar considerations apply.

Thus, the membrane proximal and transmembrane regions of gp41 carry several potential interaction sites for cholesterol. The cumulated data suggest that these interactions are relatively weak and interactions are reversible. Thus, different sites compete with each other and with oligomerization interactions within the gp41 transmembrane helix. However, even in the absence of strong, direct molecular interactions cholesterol can have regulatory, structural and dynamic effects on the membrane-embedded protein by changing the physico-chemical properties of the bilayer^1^. Recent work by the Tamm group has established that the gp41 fusion peptide requires the coexistence of L_o_/L_d_ phases where the line tension at the lipid domain boundaries contributes significantly to overcome the energetic barriers of fusion^20^. The need for cholesterol to assure efficient fusion of viral and cellular membranes^12–14^ could thereby be associated with the coexistence of L_o_ and L_d_ domains, rather than strong direct interactions.

### Structural transitions during the fusion process

An extended helix made of residues of the MPER and the gp41 transmembrane domain was observed in this paper as well as in a previous investigation^43^ (Fig. 7A). In this arrangement cholesterol induces negative curvature strain to the lipid bilayer, a feature that favors the formation of a stalk during the initial stages of the fusion process^1^. It remains possible that during the membrane fusion events a kink between the MPER and the TMD forms^44^ which allows the MPER to insert into the membrane interface^29^. In this configuration the MPER, similar to other amphipathic helices, would exert considerable positive curvature strain^29,70^ a feature that stabilizes fusion pores^1^. Thereby, the dynamic nature of the MPER-TMD where different conformations are transiently adopted has the potential to optimize the fusion process by modulating the physical properties of the membrane (Fig. 7B). The lipid interactions, the fusion peptide and the heptadrepeat domains (Fig. 1) have also been postulated to have an active role during the fusion events^31,32,88–90^. Thus, during the various intermediate stages of the fusion process not only different domains of the gp41 protein may be involved but the processes are also associated with different conformations of the MPER that have been observed in structural investigations^29,43,44^.

## Supplementary Information

Supplementary Information

## Abbreviations

AIDS: Acquired immune deficiency syndrome
CARC: Reversed CRAC
CRAC: Cholesterol recognition amino acid consensus
CCM: Cholesterol consensus motif
CD: Circular dichroism
DHPC: 1,2-Di-hexanoyl-*sn*-glycero-3-phosphocholine
DMPC: 1,2-Di-myristoyl-*sn*-glycero-3-phosphocholine
EPR: Electron paramagnetic resonance
ATR FTIR: Attenuated total reflection Fourier transform infrared
HIV: Human immunodeficiency virus
HPLC: High performance liquid chromatography
MALDI TOF: Matrix assisted laser desorption ionization time of flight
MPER: Membrane proximal external region
NMR: Nuclear magnetic resonance
POPC: 1-Palmitoyl-2-oleoyl-*sn*-glycero-3-phosphocholine
PRE: Paramagnetic relaxation enhancement
RDC: Residual dipolar coupling
SIV: Simian immunodeficiency virus
SSD: Sterol sensing domain
TFA: Trifluoroacetic acid
TFE: Trifluoroethanol
TMD: Transmembrane domain
