# Supplementary material for: Structure, membrane topology and influence of cholesterol of the membrane proximal region: transmembrane helical anchor sequence of gp41 from HIV
Source: Sci Rep. 2020 Dec 17;10:22278. doi: 10.1038/s41598-020-79327-6 (PMC7746737; doi:10.1038/s41598-020-79327-6)
Supplement: Supplementary file 1 — Supplementary Information [file 41598_2020_79327_MOESM1_ESM.pdf]

SUPPLEMENTARY INFORMATION

Structure, membrane topology and influence of cholesterol of the membrane proximal region - transmembrane helical anchor sequence of gp41 from HIV

Christopher Aisenbrey[1,#], Omar Rifi[1,#] and Burkhard Bechinger[1,2] *

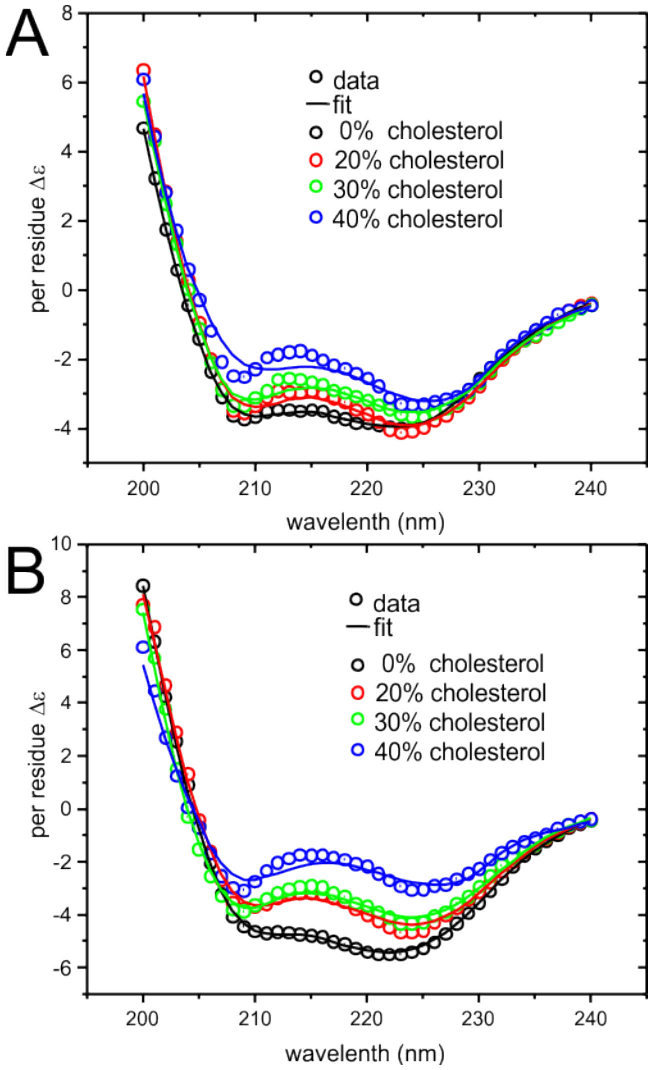

Figure S1: CD spectra shown in Figure 2A and B and comparison with the spectral deconvolutions presented in Figure 2C. The corresponding back-calculated spectra are shown as solid lines. **A.** CRAC-TM_model and **B.** CRAC-TM_gp41 after reconstitution into POPC/POPS 3/1 mole/mole SUVs with increasing concentrations of cholesterol at peptide-to-lipid ratio of 1 mole%.

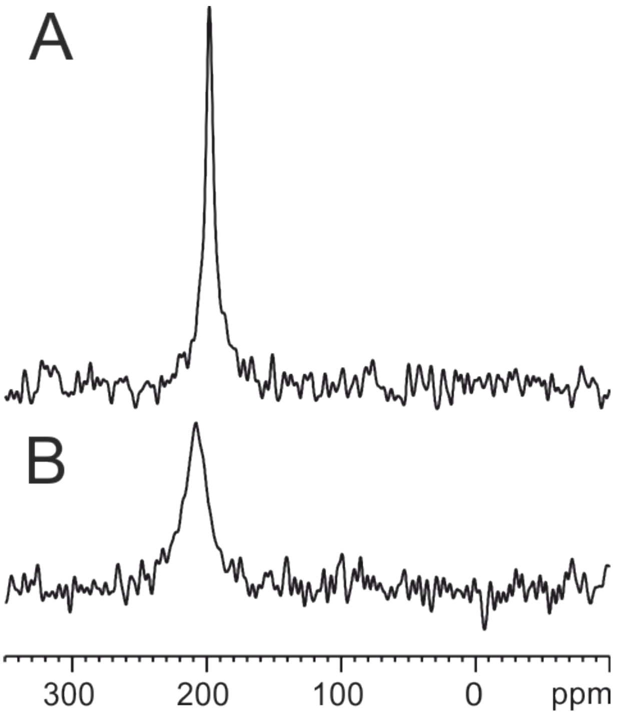

Figure S2: Proton-decoupled ^{15}N solid-state NMR spectra of 2% CRAC-TMD_model2 carrying an I-L substitution (Table 1) reconstituted into uniaxially oriented bilayers with the normal parallel to the magnetic field direction. The bilayers were made of POPC (A) or POPC/ cholesterol 70/30 mole/mole (B). In contrast to Figure 4 the presentation was chosen that both signals integrate to the same intensity thus the effect of line broadening in more apparent.

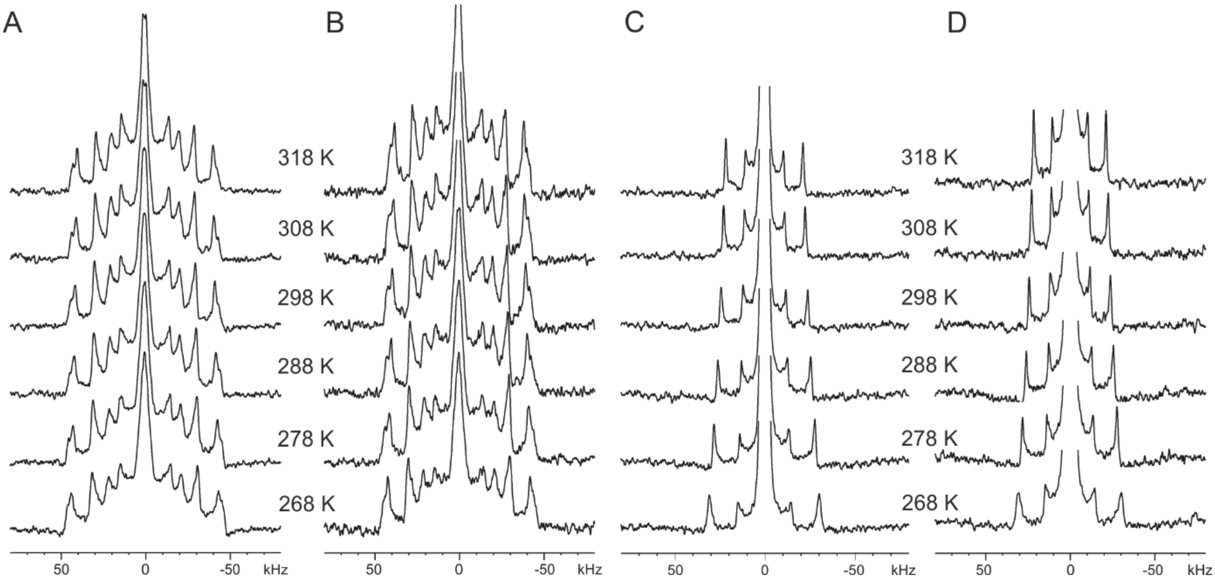

Figure S3: Quadrupolar echo deuterium spectra of glass-plate supported membranes containing cholesterol-d_6 (A,B) of cholesterol-d_7 (C,D). The membranes consist of POPC/cholesterol 90/10 mole/mole in the absence (A,C) or presence of 1 mole% CRAC-TM model (B, D). Contributions from an unoriented fraction of the sample appear with half the quadrupolar splitting.

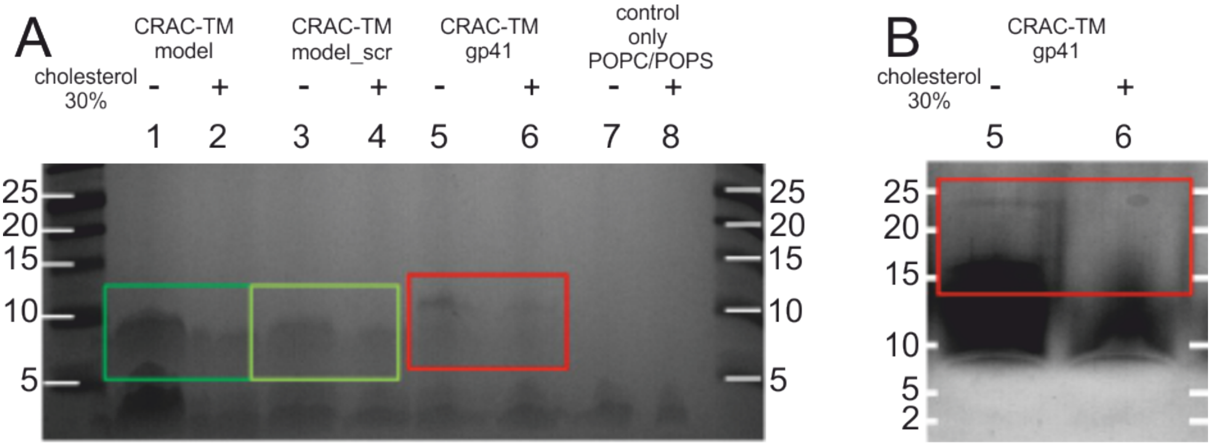

C

	monomer	dimer	trimer	dimer of trimer
CRAC-TM model	3774	7549	11324	
CRAC-TM gp41	3879		11639	23277

Figure S4: SDS PAGE of CRAC-TMD_model, its scrambled sequence and CRAC-TMD_gp41 reconstituted into POPC/POPS 3/1 mole/mole or in the presence of 30 mole% cholesterol. The samples were applied to a 4% concentration gel and separated on a 20% polyacrylamide gel. The SDS concentration was 8%. After 1h migration at 150 V the gels were stained with **A.** Coomassie (1.3 – 2 μg protein) or **B.** silver nitrate (0.5 μg protein). The marker lane is from Precision Plus Protein TM Dual Color Standards 161-0374. **C.** Theoretical molecular weight of different oligomers. The full gels are shown in Figures S5 and S6. The presentation of gel B has been stretched for easier comparison with gel A.

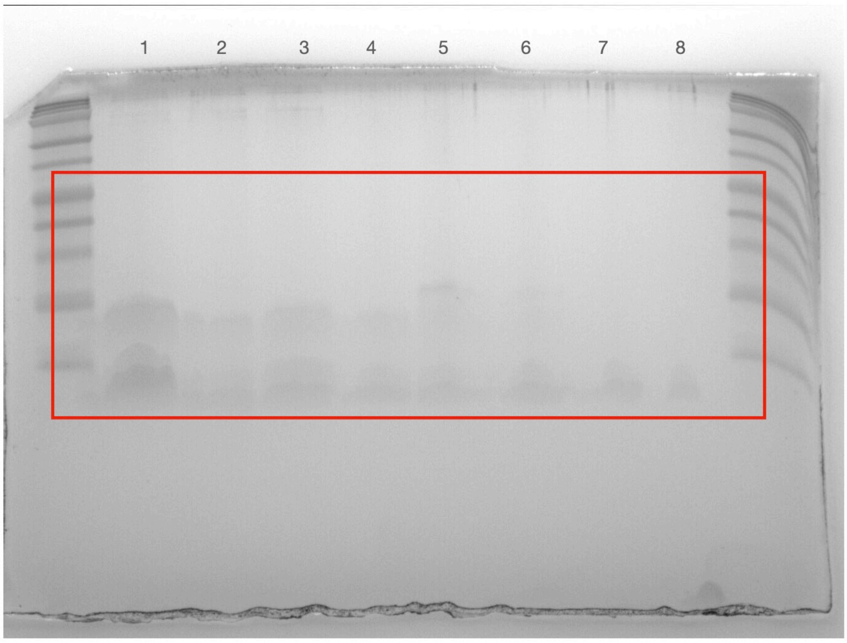

Figure S5: shows the uncropped SDS PAGE gel of which the red boxed area is analyzed in Figure S4A. A different contrast is chosen to reveal supplementary information.

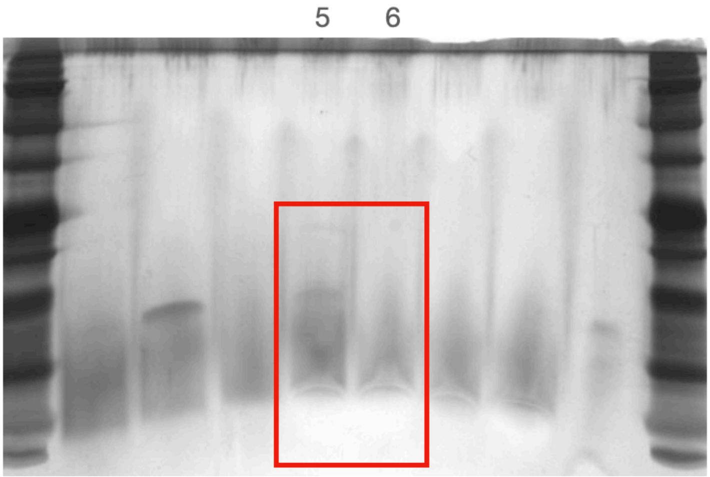

Figure S6: shows the uncropped SDS PAGE gel of which the red boxed area is analyzed in Figure S4B. A different contrast is chosen to reveal supplementary information.
